# Axonal degeneration induces distinct patterns of phosphatidylserine and phosphatidylethanolamine externalization

**DOI:** 10.1038/s41420-021-00641-7

**Published:** 2021-09-17

**Authors:** Hannah Faris, Mohammadali Almasieh, Leonard A. Levin

**Affiliations:** 1 Department of Ophthalmology and Visual Sciences, McGill University, Montreal, Canada; 2 Department of Neurology and Neurosurgery, McGill University, Montreal, Canada

**Keywords:** Cell death in the nervous system, Neurodegeneration

## Abstract

Axonal degeneration is a common feature of multiple neurodegenerative diseases, yet the mechanisms underlying its various manifestations are incompletely understood. We previously demonstrated that axonal degeneration is associated with externalization of phosphatidylserine (PS), which precedes morphological evidence of degeneration, is redox-sensitive, and is delayed in Wallerian degeneration slow (Wld^S^) mutant animals. Phosphatidylethanolamine (PE) is the other major membrane phospholipid in the inner leaflet of the cell membrane, and given that PS signals apoptosis, phagocytosis, and degeneration, we hypothesized that PS and PE membrane dynamics play distinct roles in axonal degeneration. To test this hypothesis, axonal degeneration was induced with calcium ionophores in postnatal rat retinal ganglion cells, and PS- and PE-specific fluorescent probes used to measure their externalization over time. In untreated cells, cell-surface PS was prominent in the cell body alone. Elevation of intracellular calcium with calcium ionophores resulted in significantly increased levels of PS externalization in the cell body, axon, and axon growth cone. Unlike PS, cell-surface PE was diffusely distributed in untreated cells, with comparable levels across the soma, axons, and axon terminals. After exposure to calcium ionophores, PE externalization significantly increased in the cell body and axon. Elevated intracellular calcium also resulted in the formation of axonal blebs which exclusively contained externalized PS, but not PE. Together, these results indicated distinct patterns of externalized PS and PE in normal and degenerating neurons, suggesting a differential role for these phospholipids in transducing neuronal injury.

## Introduction

**Fig. 1:**
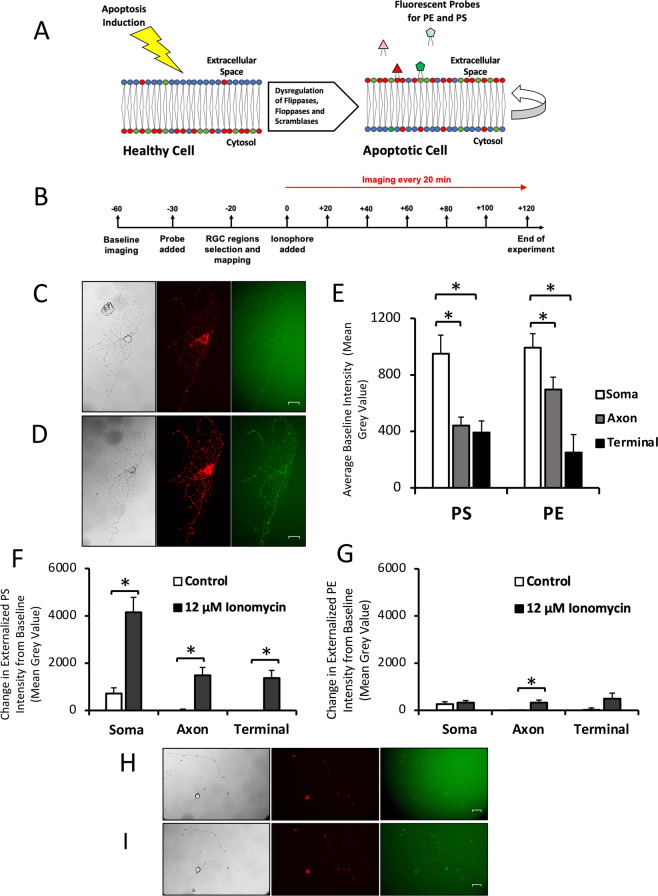
Qualitative and quantitative changes in phospholipid externalization in cultured RGCs. **A** Externalization of PS and PE during apoptosis. PS and PE are preferentially distributed in the inner leaflet of the plasma cell membrane of healthy cells. After apoptosis induction, membrane proteins are dysregulated, leading to externalization of PS and PE and a loss of membrane asymmetry. Externalized PS and PE are targets for imaging probes. **B** Timeline for cell imaging. Units are in minutes, with *t* = 0 the time point that ionophore or control media was added. The imaging probe mixture contained duramycin-GFP for imaging PE and annexin A5 for imaging PS. **C** Retinal ganglion cell before exposure to 12 uM ionomycin. Cells were in culture for 6 days prior to the experiment. Left: DIC image of cell. Middle: 670 nm image of cell, labeling PS externalization. Right: 519 nm image of cell, labeling PE externalization. **D** Retinal ganglion cell after 2 h exposure to 12 uM ionomycin. Left: DIC image of cell. Middle: 670 nm image of cell, labeling PS externalization. Right: 519 nm image of cell, labeling PE externalization. **E** Mean baseline PS and PE externalization in 20 RGCs for each cell compartment. Three measurements per cell were taken of each compartment. *p < 0.0001. **F** Change in PS externalization relative to baseline in 10 RGCs after 2 h exposure to 12 uM ionomycin or control media. For each cell 3 measurements were taken of each compartment. *p < 0.0001. **G** Change in PE externalization relative to baseline in 10 RGCs after 2 h exposure to 12 uM ionomycin or control media. For each cell 3 measurements were taken of each compartment. *p = 0.002. **H** Retinal ganglion cell before exposure to additional control media. Cells were in culture for 8 days prior to the experiment. Left: DIC image of cell. Middle: 670 nm image of cell, labeling PS externalization. Right: 519 nm image of cell, labeling PE externalization. **I** Retinal ganglion cell after 2 h exposure to additional control media. Left: DIC image of cell. Middle: 670 nm image of cell, labeling PS externalization. Right: 519 nm image of cell, labeling PE externalization. All scale bars 20 µm.

Cell death is characterized by biochemical changes in all cell compartments, including events at the plasma membrane. In healthy neurons there is an asymmetric distribution of phospholipids in the plasma membrane bilayer, with phosphatidylserine (PS) and phosphatidylethanolamine (PE) mostly concentrated in the inner leaflet, and much less in the outer leaflet [1]. The asymmetric distribution of PS and PE is maintained by lipid transporters that move phospholipids between the membrane leaflets [2]. In the early phase of cell death, altered regulation of these lipid transporters results in the loss of membrane asymmetry and externalization of phospholipids such as PS and PE into the outer leaflet of the membrane (Fig. 1A) [3].

Probes that bind PS, e.g., annexins A5 [4] and B12 [5], have enabled studies of PS externalization, including live imaging of neurons undergoing apoptosis [5, 6]. PS externalization is an early apoptotic marker in a wide variety of cell types [2, 7–10]. PS externalization serves as a recognition signal for macrophages, allowing for engulfment and disposal of somas and axons [11].

In contrast, development of non-cytotoxic imaging probes for PE has been challenging. The main probe used to image PE is duramycin, a lantibiotic peptide derived from *Streptoverticillium cinnamoneus*, which binds PE with high affinity and specificity [12]. Duramycin has been used to image PE externalization in vitro and in vivo [3]. PE externalization occurs in multiple cell types, including hypoxic or irradiated aortic endothelial cells [8] and ischemic cerebral tissue [10]. PE externalization during dynamic changes to the membrane include membrane fusion and formation of the cleavage furrow during cytokinesis [1]. PE-binding proteins (PEBPs) regulate multiple signaling pathways [13], and PEBP1 is downregulated in axotomized RGCs, while overexpression promotes axonal regeneration and survival [14].

We previously found that axonal injury to retinal ganglion cells (RGCs) results in a wave of propagation of PS externalization that travels proximally and distally along the axon from the site of injury [5]. Subsequent studies demonstrated that PS externalization and axonal degeneration can be dissociated [15]. Simultaneous imaging of PS and PE externalization has the potential to identify differences in their regulation, purpose, and distribution in injured cells. We hypothesized that externalization of the more abundant PE might be considerably different than PS, particularly with respect to PE’s role in structural membrane events [16–18]. To test this hypothesis, we performed longitudinal imaging of PS and PE in primary cultures of RGCs undergoing axonal degeneration induced by elevated intracellular calcium levels. We found distinct patterns and time courses for externalized PS and PE at baseline and in degenerating RGCs. Surprisingly, we identified highly localized PS but not PE in blebs (bulb-like excrescences of the membrane [19]), suggesting a role for these phospholipids in regulating the shape of microdomains in degenerating axons.

## Results

### Differential compartmentalization of externalized phosphatidylserine and phosphatidylethanolamine in retinal ganglion cells

At baseline, most RGCs expressed some externalized PS (Fig. 1C, H), likely reflecting the shock associated with the addition and removal of cell media and temperature and atmospheric transitions when culture dishes are moved from the incubator to the microscope microincubator. There was a significant difference (*p* < 0.0001 by one-way ANOVA) in the level of externalized PS across the 3 cellular compartments analyzed (cell body, axon, and axon terminals) (Fig. 1E). Pairwise post hoc comparisons showed a significantly greater level of externalized PS between the cell body and the axon (1170 ± 160 vs. 441 ± 60; *p* < 0.0001); and cell body and axon terminals (1170 ± 160 vs. 392 ± 82; *p* < 0.0001) but not between the axon and axon terminals (*p* = 0.945).

There was a small amount of PE externalization observed across all cell compartments at baseline (Fig. 1C), with a significant (*p* < 0.0001) difference in the level of externalized PE across cell compartments (Fig. 1E). Post hoc testing showed a significant difference between the cell bodies and axon terminals (993 ± 99 vs. 250 ± 127; *p* < 0.0001), between the axon and axon terminal (695 ± 88 vs. 250 ± 127; *p* < 0.0001), but not between the cell body and axon (*p* = 0.12). PE externalization was associated with a brighter and more heterogeneous background than PS externalization. Overall, PE externalization differed across cell compartments, with a greater level of PE externalization observed in the cell body than axon terminals. However, compared to PS externalization, the differences in PE externalization between compartments were less (Table 1).

**Table 1:** Qualitative differences in phospholipid externalization in different cell regions at baseline.

Baseline externalization			
	Cell body	Axon	Axon terminal
**PS**	+ + +	+	+
**PE**	+	+	−

Plus signs represent increases in phospholipid externalization, and a minus sign represents no change in externalization.

### Elevated intracellular calcium induces distinct spatial distributions of externalized phosphatidylserine and phosphatidylethanolamine

The calcium ionophore ionomycin (8 and 12 µM) was used to elevate intracellular calcium levels. When comparing the change from baseline in PS externalization in cells incubated for 2 h with ionomycin relative to control cells, the levels of externalized PS significantly increased (Fig. 1F) in all three compartments: cell body (4154 ± 626 ionomycin vs. 720 ± 237 control; *p* < 0.0001); axon (1478 ± 341 vs. −6 ± 62; *p* < 0.001); and axon terminals (1372 ± 322 vs. −55 ± 67; *p* < 0.0001). The intensity in the compartments increased by several times the baseline level in all compartments, indicating a large increase in PS externalization across the entire cell. In contrast, control cells had exhibited only a modest increase in PS externalization after 2 h (Fig. 1I).

In cells exposed to ionomycin, there was a significantly greater increase in PS externalization in the cell body compared to the axon (4153 ± 626 vs. 1478 ± 341; *p* = 0.0001), and cell body compared to the axon terminal (4152 ± 626 vs. 1372 ± 322; *p* = 0.0002), but not the axon relative to the axon terminal (*p* = 0.985). Control cells also had a significantly greater increase in PS externalization in the cell body compared to the axon (721 ± 237 vs. −6 ± 62; *p* < 0.001), and cell body compared to axon terminal (721 ± 237 vs. −55 ± 67; *p* < 0.001), but not the axon relative to the axon terminal (*p* = 0.970). Overall, ionomycin led to an increase in PS externalization across the entire cell, with the greatest increase in PS externalization observed in the cell body. Similar results were observed when intracellular calcium was elevated with A23187.

When comparing the changes in PE externalization in cells incubated with ionomycin for 2 h relative to control cells (Fig. 1G), the levels of externalized PE significantly increased in the axon (325 ± 109 vs. −139 ± 93; *p* = 0.002) but not the cell body (*p* = 0.63) or axon terminal (*p* = 0.07). There was no difference among cell compartments in PE externalization after 2 h incubation with ionomycin (*p* = 0.71). However, in the control condition the change in PE externalization was significantly different in the cell body relative to the axon (261 ± 101 vs. −139 ± 93; *p* = 0.01); but not between the cell body and axon terminal (*p* = 0.16), or axon and axon terminal (*p* = 0.50). Overall, after 2 h incubation with ionomycin there was a similar increase in PE externalization across all cell compartments (Table 2), but only the change in the axons was statistically significant. Note that the heterogeneous intensity observed in the PE images at baseline led to more variability in the quantitative measurements of intensity of PE externalization.

**Table 2:** Qualitative change in phospholipid externalization compared to control cells after 2 h exposure to 12 µM ionomycin.

Change in externalization relative to control after exposure to 12 µM Ionomycin			
	Cell body	Axon	Axon terminal
**PS**	+ + +	+	+
**PE**	−	+	−

Plus signs represent increases in phospholipid externalization, and minus signs represent no change in externalization.

### Exposure to calcium ionophores leads to formation of membrane blebs that contain externalized PS but not PE

There were prominent morphological changes in RGCs after exposure to calcium ionophores, with most RGCs demonstrating swelling of the cell body, accompanied by axonal beading, blebbing, and degeneration (Fig. 1D). Exposure to ionomycin or A23187 eventually led to neuronal degeneration, with blebs forming in the axons. By the end of the observation period (120 min) these blebs appeared in 71.9% (41/57) of all RGCs treated with an ionophore, with a total of 453 blebs counted across those 41 cells, and each RGC expressing a mean of 11 ± 2 blebs. In the absence of ionophore, only 11.5% (3/26) of the cells had developed blebs, and each positive cell only expressed a single bleb. The bleb formation patterns and numbers were similar between ionomycin and A23187. The overall breakdown of the number of blebs observed across each condition is summarized in Table 3.

**Table 3:** Summary of bleb data across cell conditions after 2 h exposure to calcium ionophore or control media.

Condition	Number of cells	# Positive cells	# Blebs	Mean # blebs in positive cells	Cells with blebs (%)
**Control**	26	3	3	1.0	11.5
**8** **µM ionomycin**	15	8	27	3.4	53.3
**12** **µM ionomycin**	32	24	313	13.0	75.0
**12** **µM A23187**	10	9	113	12.6	90.0
**All ionophore-treated cells**	57	41	453	11.0	71.9
**Total**	83	44	456	10.4	53.0

**Fig. 2:**
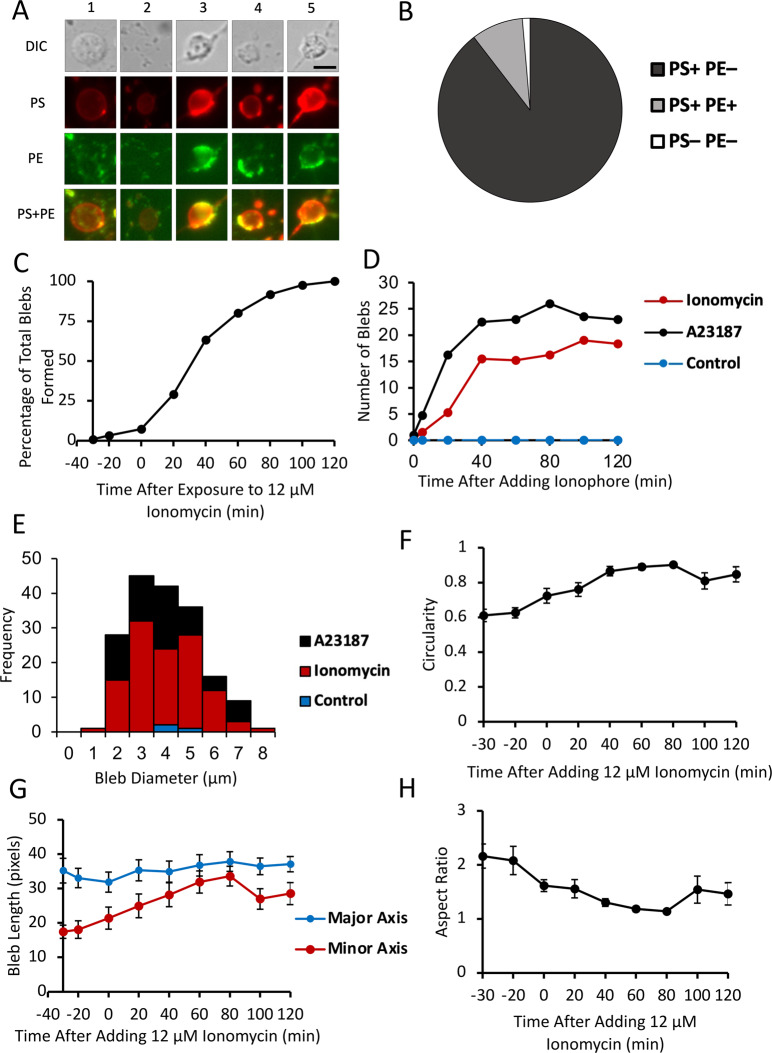
Morphological characteristics, time dependence, and geometric aspects of bleb formation. **A** Examples of blebs with different staining patterns. 1: PS + PE–; 2: PS + PE– Para-axonal PE + ; 3: PS + Half of Bleb PE + Para-axonal Staining + ; 4: PS + Half of Bleb PE + Para-axonal Staining–; 5: PS + PE + . Scale bar 5 µm. **B** Classification of all blebs (*n* = 220) observed in 5 cells exposed to 12 µM ionomycin, based on staining of externalized PS and PE. **C** Proportion of blebs formed before and after exposure to 12 µM ionomycin. **D** Mean number of axonal blebs in selected RGCs after addition of calcium ionophore. Number of blebs counted for four cells of each treatment. Cells were exposed to A23187, ionomycin, or control media. **E** Size distribution of RGC axonal blebs. Bleb diameter was measured for all blebs in 2 cells for each condition: control, ionomycin, and A23187. Measurement taken in cells after 2 h exposure to calcium ionophore or control media. Mean diameter of blebs was 3.45 μm. **F** Mean circularity of 10 blebs exposed to 12 uM ionomycin over time; 1 is equivalent to a perfect circle. **G** Mean length of 10 blebs along its major and minor axes before and after exposure to 12 µM ionomycin. **H** Mean aspect ratio of 10 blebs before and after exposure to 12 µM ionomycin. Aspect ratio was calculated by dividing the maximum axis by the minimum axis length.

Most blebs had a distinctive pattern of externalized PS staining (Fig. 2A). Typically, the circumference of the bleb strongly labeled for externalized PS, with the interior of the bleb having a lower level of PS externalization compared to background. The distribution of externalized PS likely reflects the circular cross-section of a spherical shape imaged as a two-dimensional image. This pattern was recapitulated across all conditions, including incubation with both ionomycin and A23187. The finding that different calcium ionophores, acting via different intracellular mechanisms, both resulted in bleb formation suggests that increased intracellular calcium alone is enough to induce PS-containing bleb formation. Of 220 blebs observed in 5 cells exposed to 12 µM ionomycin, 217 blebs demonstrated this PS-positive rim and interior pattern and 3 blebs had no externalized PS or PE (Fig. 2B).

Several patterns of PE externalization in blebs were observed (Fig. 2A). Of the 217 blebs observed in cells exposed to 12 µM ionomycin which had externalized PS, 97 (44.7%) had no externalized PE staining at all, 112 (51.6%) had no PE staining of the bleb but did have para-axonal PE staining proximal or distal to the bleb, 9 (4.1%) had no para-axonal PE staining but did have at least half of the bleb stained with PE, 6 (2.8%) had para-axonal PE staining where at least half of the bleb stained with PE, and 10 (4.6%) blebs were fully positive for PE. The pattern of PE staining was not associated with the timing of when the blebs formed (*p* = 0.15) or when they collapsed (*p* = 0.18). While para-axonal staining was frequently observed, there was no association between para-axonal staining and bleb characteristics such as formation time (*p* = 0.15) or whether the blebs collapsed (*p* = 0.10).

The time of bleb formation after exposure to 12 µM ionomycin varied, with 50% forming by 40 min and 90% by 80 min (Fig. 2C). The number of blebs present in an axon plateaued about 40 min after exposure to ionomycin or A23187 (Fig. 2D). This likely reflects an equilibrium of bleb formation and collapse after this time point. There was a significant difference in time of bleb formation, depending on whether they formed in unbranched axons, the branchpoints of axons, or axon terminals. Blebs at terminals formed later than blebs at branchpoints (58.3 ± 6.4 vs. 40.1 ± 2.8 min; *p* = 0.015), but there was no significant difference between time of formation between blebs at terminals and unbranched axons (*p* = 0.235) or between blebs at branched versus unbranched axons (*p* = 0.14).

Forty-two of 220 blebs (19.1%) collapsed at some point during observation, with 50% of those blebs collapsing by 80 min exposure to 12 µM ionomycin, and 75% of those blebs collapsing by 100 min exposure to 12 µM ionomycin. There was no significant difference on whether a bleb collapsed (*p* = 0.38) or the time of collapse (*p* = 0.15) with respect to whether the bleb was localized to the terminals, unbranched axons, or axon branchpoints. When a bleb collapsed, the characteristic externalized PS pattern with the stained rim and interior was lost, leaving a smaller bead-shaped bleb on the axon with uniform PS staining. Blebs that did not collapse grew over time and then stabilized to a uniform size by the end of the recording session (120 min).

The mean bleb diameter was 3.45 ± 0.10 µm (Fig. 2E), and was not normally distributed (*p* = 0.01 by Kolmogorov–Smirnov test) because it was skewed towards larger blebs. Blebs were nearly circular, with a circularity of 0.925 ± 0.003 (Fig. 2F), and an aspect ratio of 1.09 ± 0.01 (Fig. 2G–H). As blebs formed and grew, the circularity increased over time. If a bleb collapsed, circularity was lost.

**Fig. 3:**
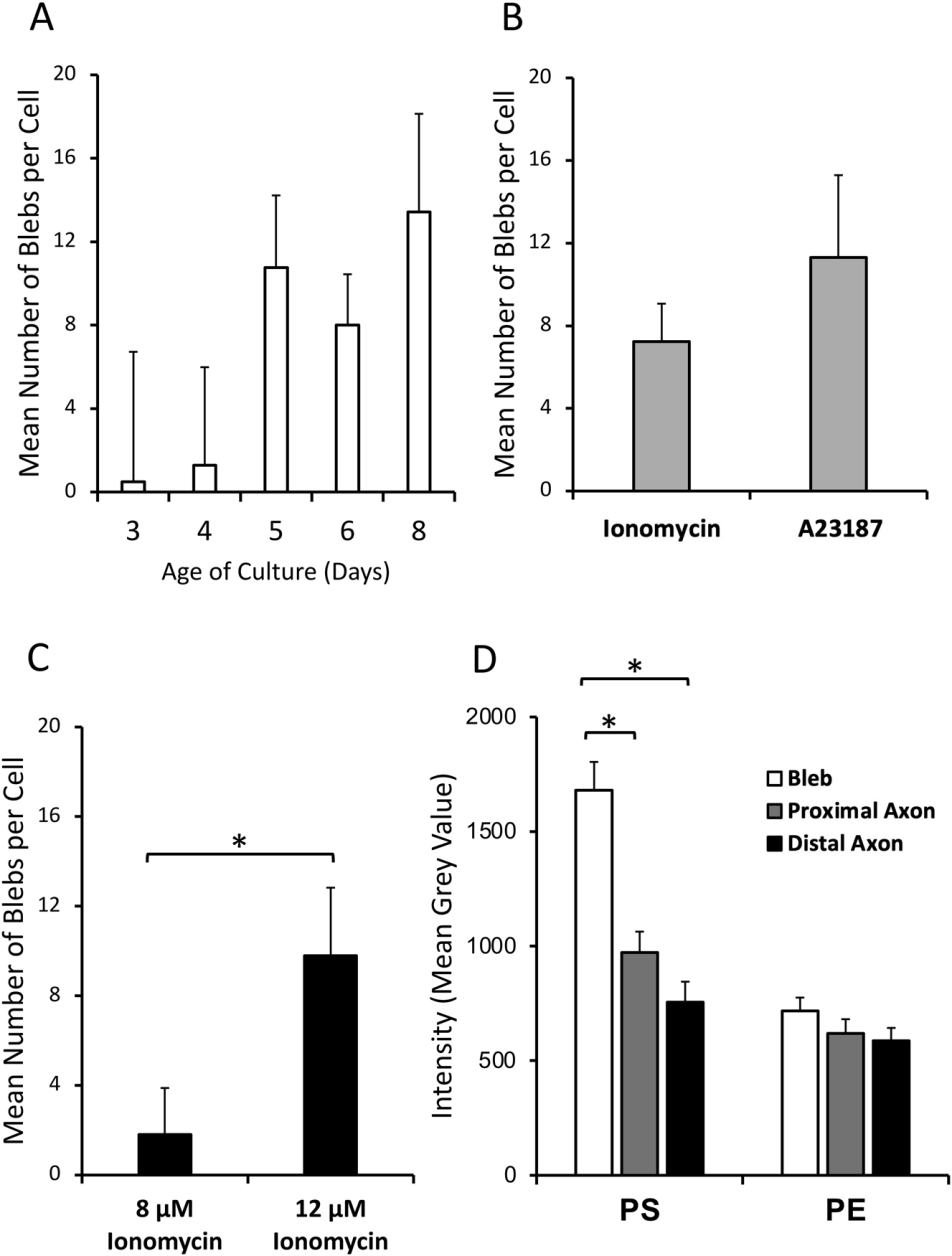
Culture-time dependence, ionophore selectivity, and axonal region intensity of PS and PE externalization. **A** Mean number of blebs per cell after 2 h exposure to ionophore, by age of cell culture in days. **B** Mean number of blebs per cell after 2 h exposure to ionophores. **C** Mean number of blebs per cell after 2 h exposure to ionomycin. **D** Difference in intensity of bleb compared to adjacent axon regions. The intensity of 5 blebs from 10 cells each were measured, with three intensity measurements each for the bleb, proximal axon, and distal axon. *P < 0.001.

There were no significant differences in the mean number of blebs per cell present after 2 h of ionophore incubation with respect to either the age of culture at the time of the experiment (*p* = 0.26; Fig. 3A) or which calcium ionophore (ionomycin or A23187) was used (7.2 ± 1.8 vs. 11.3 ± 4.5; *p* = 0.36; Fig. 3B). There was a significant effect of ionophore concentration on the number of blebs formed, with 12 µM associated with formation of more blebs per cell than 8 µM (9.8 ± 3.0 vs. 1.8 ± 2.0; *p* = 0.036; Fig. 3C).

There was a significant difference in the degree of PS externalization (captured as fluorescence intensity) in blebs compared to their adjacent proximal and distal axon segments (*p* < 0.001; Fig. 3D). Specifically, PS externalization was significantly different between blebs and their proximal axon (1680 ± 124 vs. 972 ± 91; *p* < 0.001) or distal axon (1680 ± 124 vs. 756 ± 89; *p* < 0.001), but not between the proximal and distal axon (972 ± 91 vs. 756 ± 89; *p* = 0.30). In contrast, for PE there was no minimal difference in fluorescence intensity between blebs and their proximal or distal axonal regions (*p* = 0.27; Fig. 3D). Together, this indicates increased levels of externalized PS but not PE in blebs relative to their adjacent axonal segments.

## Discussion

We used calcium-induced axonal degeneration in cultures of purified RGCs as a model for studying PS and PE externalization in neuronal membranes. We used two different calcium ionophores to trigger axonal degeneration, each increasing intracellular calcium levels through different mechanisms (ionomycin via release from intracellular calcium stores [20] and A23187 via extracellular calcium entry [21]). The annexin A5 probe used for detection of PS is commercially available and provides a strong signal with minimal background, enabling precise detection of changes in fluorescence [22]. For detection of PE, we used a non-cytotoxic duramycin probe containing a polyethylene glycol linker to GFP, enabling a reliable method for imaging PE compared to previous methods [1].

We previously demonstrated a redox-dependent mechanism for PS externalization in RGC axons after axotomy [5] and hypothesized that the same would be true for PE, given that externalization of PS and PE is usually synchronized in neuronal and non-neuronal cells [2, 3, 10]. It was therefore surprising to find a significant difference in the pattern of PS and PE externalization in RGCs associated with axonal degeneration induced by calcium ionophores. PS externalization was primarily localized at the soma and axon terminals, whereas PE externalization was more uniformly distributed across the cell body, axon, and axon terminals, with significant externalization in the axon. In addition, PS externalization was localized in blebs, whereas PE externalization was not observed in blebs. Despite the differences in mechanism of calcium release by the two ionophores, both caused region-specific phospholipid externalization patterns that were specific for PS and PE. This suggests that increased intracellular calcium is sufficient to induce region-specific PS and PE externalization in neurons. Although duramycin only binds to PE, annexin A5 may bind PE and phosphatidylinositol to a small degree [2]. However, if off-target binding of annexin A5 to PE were occurring, then it would not explain the absence of duramycin binding at blebs nor other disparities between annexin A5 and duramycin binding.

The type of injury can affect the pattern of PS and PE of externalization in membranes during apoptosis [8]. Yet other studies have not shown obvious differences in the spatial patterns of PS and PE externalization, and therefore our finding of distinctive patterns for PS and PE in calcium-induced axonal degeneration may be relevant to the mechanisms of signaling of axonal injury [5]. Duramycin more strongly binds PE-containing liposomes that are smaller and with higher curvature than those that are larger and have lower curvature [23]. The differential distribution of PS and PE externalization in RGC neuronal compartments may similarly relate to their dimensions, and specifically the degree of membrane curvature. PE forms non-lamellar membrane structures, enhancing and changing membrane curvature, while its cone shape structure [18] affects membrane dynamics such as fusion/fission [17], cytoskeletal organization, and cell polarization [16]. We observed large differences in PS externalization between cell body and axon (Fig. 1F). In comparison, there were much smaller differences in PE externalization between cell body and axon. Cell bodies have large diameters and hence small curvatures, while axons have very small diameters and therefore high curvatures. We hypothesize that PS externalization is greater with lower curvature, but PE externalization is not. Supporting this are data from PS and PE externalization in blebs. Geometry implies that regions of axons where there is beading and blebbing have locally increased axonal diameters, and therefore decreased curvatures. Indeed, PS externalization is greater in blebs, compared to adjacent axon segments, but this was not true for PE. Dynamic changes in membrane curvature could therefore explain regional differences in the degree of PS and PE externalization.

The physical dimensions of the imaging probes are unlikely causes of regional differences in PS and PE externalization. Alexa Fluor 647-conjugated annexin A5 is similar in molecular weight (36 kDa) to duramycin-GFP and the sizes of the imaging probes are approximately 3-4 nm [24], 3 orders of magnitude smaller than axonal diameters and 2 orders of magnitude smaller than blebs.

The abundance of externalized PS at the cell body may arise from high levels of PS in nuclear and endoplasmic reticulum membranes [25]. This provides a ready source for translocation of PS-containing vesicles from ER to the RGC plasma membrane after exposure to calcium ionophores. At baseline there were also different patterns of PS and PE externalization in RGCs. Primary RGCs are not static but extend axons and growth cones. The presence of PS at the growth cones and axonal terminals of RGCs may reflect its contribution to dynamic membrane changes associated with formation of filopodia and axon growth [26, 27].

This study focused on calcium-induced loss of membrane polarity. Elevated intracellular calcium is a critical factor in initiation of axonal degeneration: axotomized axons in calcium-deficient media have delayed degeneration [28]; increased survival of RGCs exposed to optic nerve crush occurs when calpain activity is reduced with calcium channel inhibitors [29], and calcium ionophores exacerbate axonal degeneration in lesioned axons [19]. Both ATP-binding cassette (ABC) proteins (responsible for the outward translocation of phospholipids) and phospholipid scramblases are activated by elevated calcium levels, leading to both PS and PE externalization and consequent loss of membrane asymmetry [2].

### Phospholipid externalization is differentially associated with the formation of axonal blebs

RGCs incubated with calcium ionophores developed morphological changes in axons characteristic of axonal degeneration, initially with the formation of bleb-like structures along the length of the axon, and eventually proceeding to axonal beading. Membrane blebs are plasma membrane regions which expand and protrude beyond the cell cytoskeleton, commonly occurring during apoptosis and necrosis [30]. Blebs originated from areas of the axonal membrane externalizing PS, while axonal segments surrounding blebs externalized PE.

The greater levels of externalized PS in blebs relative to PE could be due to a more critical role for cytoplasmic PS than PE in maintaining the connection between the axonal membrane and cytoskeleton, so that reduction of PS at inner leaflet would contribute more profoundly to bleb formation [31]. Alternatively, PS externalization in blebs may be an early step for providing a signal to macrophages to begin engulfing that part of the axon. Finally, the physical characteristics of the bleb, such as its thickness or curvature, could cause PS to be preferentially concentrated in blebs.

Phospholipid translocation in blebs occurs in non-neuronal cells such as apoptotic tumor cells [8] (PS and PE) and cytotoxic T cells [32] (PE). Differences of those results from RGC axons could reflect the axonal microenvironment, the PE imaging probe, or the nature of injury.

It is not surprising that PS and PE translocation are involved in bleb formation because their externalization is an indication of loss of membrane asymmetry. Loss of membrane polarity loosens connections between actin and fodrin with the plasma membrane [33], and destabilizes microtubules and actin-myosin II interactions [34] allowing the intracellular pressure to form and expand blebs [2]. Elevated intracellular calcium also activates calpains, breaking actin filaments and hydrolyzing fodrin attachments of actin to the cell membrane [30], inducing cytoskeletal degradation and axonal degeneration. Elevated cytoplasmic calcium levels may also contribute to microtubule disassembly [19] by interacting with inner-leaflet phospholipids [35].

In summary, we demonstrated spatially distinct patterns of PS and PE externalization in calcium-dependent axonal degeneration and axon bleb formation in RGCs. Understanding how membrane translocation of these phospholipids is regulated will help in understanding the dynamics of axonal degeneration and could be useful in developing membrane targets for axonal protection in neurodegenerative diseases.

## Methods

### Primary cell culture

Purified RGC cultures were prepared from dissociated Sprague Dawley P2-P7 rat retinas of either sex that were immunopurified by sequential anti-macrophage and anti-Thy-1 panning [5]. All animal experiments were conducted in accordance with the guidelines of the Canadian Council on Animal Care and approved by the Montreal Neurological Institute and McGill University Animal Ethics Committees.

### Incubation, staining, and imaging procedures for primary culture experiments

Time-lapse video recording of RGCs used duramycin-GFP and annexin A5, imaged with differential interference contrast (DIC) and filters for GFP and Cy5, respectively, before and after addition of calcium ionophores ionomycin and A23187. Figure 1B summarizes the timeline for each experiment. Supplementary Video 1A–C depicts an example of a single cell imaged in the DIC, GFP (duramycin), and Cy5 (annexin A5) channels, respectively.

### Data analysis

Images from experiments were analyzed with cellSens Dimension software (Waltham, MA), and ImageJ (Bethesda, MD). Graphical analysis was performed with Microsoft Office Excel (Redmond, WA), and statistical analysis was performed with JMP (Cary, NC).

## Supplementary information

Supplementary Methods

Supplementary Video 1A

Supplementary Video 1B

Supplementary Video 1C

## Data Availability

The datasets analyzed during the current study are available from the corresponding author on reasonable request.
