# Supplementary material for: Axonal degeneration induces distinct patterns of phosphatidylserine and phosphatidylethanolamine externalization
Source: Cell Death Discov. 2021 Sep 17;7:247. doi: 10.1038/s41420-021-00641-7 (PMC8448818; doi:10.1038/s41420-021-00641-7)
Supplement: Supplementary file 1 — Supplementary Methods [file 41420_2021_641_MOESM1_ESM.docx]

# **Supplementary Methods**

## Materials

Glass-bottom dishes were from MatTek (Ashland, MA). Goat anti-rabbit igG (H+L) and goat anti-mouse IgM were from Jackson ImmunoResearch (West Grove, PA). Rabbit anti-rat macrophage sera was from Cedarlane (Burlington, NC). Anti-Thy-1 IgM was produced from the T11D7e2 hybridoma line (ATCC). Poly D-lysine, DNase I, insulin, forskolin, bovine serum albumin, trypsin, trypsin inhibitor, human apo-transferrin, progesterone, sodium selenite, putrescine, thyroxine (T3), N-acetyl-L-cysteine, phenol red, and A23187 were from Sigma-Aldrich (St. Louis, MO). Cultrex mouse laminin I was from Trevigen (Gaithersburg, MD). Dulbecco’s phosphate-buffered saline (PBS), fetal bovine serum, Neurobasal-A, penicillin/streptomycin, sodium pyruvate, Earle’s balanced salt solution, and L-glutamine were from Invitrogen (Eugene, OR). Papain was from Worthington Biochemical (Lakewood, NJ). L-cysteine was from Wisent (St-Bruno, Canada). Brain-derived neurotrophic factor and ciliary neurotrophic factor were from Alomone Laboratories (Jerusalem, Israel). Recombinant human basic fibroblast growth factor (154 aa) was from PeproTech (Montreal, Canada). Defined serum supplement GS-21 was from MTI-GlobalStem (Gaithersburg, MD). Ionomycin, Alexa Fluor 647-conjugated annexin A5, and petri dishes were from Thermo Fisher Scientific (Waltham, MA). Duramycin-GFP was synthesized at the Zhao Lab at Northwestern University (Chicago IL).

## Preparation of imaging probes and treatment agents

For each experiment, 2.5 µL of Alexa Fluor 647-conjugated annexin A5 was used to achieve a final concentration of 4 µg/mL in RGC medium. Duramycin-GFP was used to identify externalized PE. Lyophilized duramycin-GFP powder was resuspended in 100 µL of RGC medium to make a 100 µg/mL stock solution and used at a final concentration of 20 µg/mL. The solution was vortexed to ensure resuspension and centrifuged at 2000 g to remove large particulate matter. Duramycin in its pure form was not used in concentrations greater than 2 µM due to its possible cytotoxicity^1^.

## Incubation, staining, and imaging procedures for primary culture experiments

Time-lapse video recording of RGCs was performed using an Olympus IX83 microscope (Waltham, MA). The imaging probe mixture (duramycin-GFP and 2.5 µL annexin A5) was added, gently swirled, and incubated for 10 minutes. Baseline microphotographs were then taken with differential interference contrast (DIC), duramycin-GFP was imaged with filters for GFP (excitation at 495 nm, emission at 519 nm). Alexa Fluor 674-conjugated annexin A5 was imaged with filters for Cy5 (excitation at 625 nm, emission at 692 nm, and dichroic at 670 nm). Baseline photos were taken before and after the addition of the imaging mixture. Then ionomycin (8 or 12 µM), A23187 (8 or 12 µM), or RGC media alone was added to the dish. These concentrations were chosen based on previous experiments that tested a broad range of calcium ionophores, finding that 8 µM and 12 µM were optimally caused cell death and phospholipid externalization over the course of a few hours. The cells of interest were then imaged every 20 minutes for 2 hours. Figure 1B summarizes the timeline for each experiment. Supplementary Videos 1A, 1B, and 1C depict an example of a single cell imaged in the DIC, GFP (duramycin), and Cy5 (annexin A5) channels, respectively.

A total of 83 RGCs were observed across 14 different experiments, utilizing cells from 4 independent primary cultures, each derived from different sets of rat pups. Sample sizes were not chosen prospectively. The control and A23187 experiments were each performed 3 times, and a total of 3 experiments using 8 µM ionomycin and 5 experiments with 12 µM ionomycin were performed. Data analysis incorporated cells across different experiments and batches. All data were analyzed and reported, and no randomization was performed. To control for the effects of manipulation of the media when adding the calcium ionophores, 5 uL of conjugated annexin A5 was added directly to the original 1.5 mL of media, followed by removing 1.4 mL of media and imaging the cells over time. There were no observable changes in cell morphology or levels of PS externalization after changing or reducing the volume of media.

## Primary cell culture

Purified RGC cultures were prepared from dissociated Sprague Dawley P2-P7 rat retinas of either sex that were immunopurified by sequential anti-macrophage and anti-Thy-1 panning^2^. All animal experiments were conducted in accordance with the guidelines of the Canadian Council on Animal Care and approved by the Montreal Neurological Institute and McGill University Animal Ethics Committees.

Briefly, freshly dissected retinas were placed in a filtered PBS solution containing 16.5 U/ml papain, 0.2mg/ml L-cysteine, 124 U/ml DNase I, and 1 mM sodium hydroxide solution. The tube containing the retinas was incubated in water bath for 30 min at 37°C, gently swirling the solution at 15 min intervals. After digestion, the papain solution was gently aspirated, retaining the partially digested retinas. Ovomucoid (0.6mg/ml) and BSA (0.6 mg/ml) mixed with anti-rat macrophage antibody were added to halt digestion and used as medium for the gentle trituration of the retinas, resulting in a single cell suspension. After 10 min to allow antibody binding, cells were pelleted by centrifugation and resuspended in PBS containing 10 mg/ml ovomucoid and BSA. Cells were pelleted again and resuspended in DBPS containing 0.2 mg/ml BSA and 5 µg/ml insulin. The cell suspension was added to a 150 mm Petri dish that had been incubated overnight with goat anti-rabbit IgG in Tris-HCl and incubated for 20 min at room temperature to allow macrophages to adhere to the plate, agitating after 10 min. Nonadherent cells were transferred to a second identical 150 mm dish and incubated for 45 min (agitating at 15 min intervals) to further deplete macrophages. The binding of macrophages was confirmed on a microscope. Nonadherent cells were then gently shaken loose and seeded onto a 100 mm Petri dish that had been previously coated with goat anti-mouse IgM in Tris-HCl, followed by supernatant from the T11D7e2 hybridoma line containing anti-Thy-1 IgM. Cells were incubated 50 min at room temperature to allow binding of RGCs, agitating every 10 min. Nonadherent cells were then removed by pipetting and the plate very gently rinsed four to six times with PBS until only adherent cells remained, as assessed by microscopy. The RGCs adherent to the anti-Thy-1 dish were removed with a trypsin incubation at 37°C for 4 min, followed by inactivation with 30% heat-inactivated FBS and dislodging by pipetting. Cells were collected in a 20 ml plastic tube, pelleted, and resuspended in 500 µl of 30% FBS for counting on a hemocytometer, at which point the quality of isolation was estimated by uniformity, shape, and size of isolated cells. The purified RGCs were plated on MatTek glass-bottom culture dishes that had been previously coated with poly-D-lysine (10 µg/ml) for at least 30 min and with laminin in Neurobasal A (2 µg/ml) overnight. The RGC culture medium consisted of Neurobasal A supplemented with 2% GS-21 (a defined serum supplement), penicillin (100 U/ml), streptomycin (100 µg/ml), insulin (5 µg/ml), sodium pyruvate (1 mM), L-glutamine (1 mM), transferrin (100 µg/ml), BSA (100 µg/ml), progesterone (0.2 µM), putrescine (16 µg/ml), sodium selenite (40 ng/ml), T3 (40 ng/ml), N-acetyl cysteine (5 µg/ml), forskolin (5 µM), BDNF (50 ng/ml), CNTF (10 µg/ml), and bFGF (10 µg/ml). Cells were kept in a humidified 10% CO_2_ incubator at 37°C for at least 3 d to allow time for the ganglion cells to adhere and extend processes and fed every 3 d by removal of half the medium and replacement with fresh medium.

## Data analysis

For the quantitative analysis of levels of PE and PS externalization across cell regions, the mean grey-scale value, a measurement of the intensity of the fluorescent labeling, was taken as a measurement of the level of externalization present in that region. For each cell, ImageJ was used to define 9 identically sized circular regions of interest, 3 each for the cell body, axon, and axon terminal. For each image, a mean background intensity was computed from 10 regions of interest selected randomly from those parts of the image that did not contain RGCs. This mean background intensity was subtracted from the measured intensity for the 9 regions of interest on the cell.

Independent measures one-way ANOVA was used to test for differences in fluorescence intensity with respect to phospholipid (PS or PE), region of cell or axon, time of imaging, and staining patterns of blebs, and timing of bleb formation or collapse. If the ANOVA was significant (p < 0.05), it was followed by Tukey’s honestly significant difference (HSD) test for pairwise *post hoc* comparison testing.

For analysis of bleb geometry, they were traced and the major and minor axes, perimeter, and area measured in order to calculate bleb aspect ratio and circularity. The Kolmogorov-Smirnov test was used to assess normality of the distribution of bleb size.

## References

1. Larson MC, Woodliff JE, Hillery CA, Kearl TJ, Zhao M. Phosphatidylethanolamine is externalized at the surface of microparticles. *Biochim Biophys Acta* 2012, **1821**(12)**:** 1501-1507.

2. Almasieh M, Catrinescu MM, Binan L, Costantino S, Levin LA. Axonal Degeneration in Retinal Ganglion Cells Is Associated with a Membrane Polarity-Sensitive Redox Process. *J Neurosci* 2017, **37**(14)**:** 3824-3839.
